# Progress in Medicine

**Published:** 1897-08-14

**Authors:** 


### Progress in Medicine.

### FEVERS.

*(Continued from page 182.)*

**Scarlet Fever.**—Few cases of the use of streptococcal serum have been recorded. In addition to those noticed previously, Knyvett Gordon[25] mentions one of a boy apparently moribund, with an offensive discharge from the fauces and general septicæmia on the eighth day of an attack. Two doses of 10 c.c. of serum were administered, and rapid recovery followed. The author suggests that the method will be useful where septicæmic symptoms or absorption from a sloughing throat cause danger. Yot[26] chronicles another case, in which improvement followed each injection, and a good recovery was made. Speransky[27] claims that arsenic acts as a preventative, and if the disease has been acquired it puts an end to the fever in 24 hours, and causes rapid recovery. He administers small doses of Fowler's solution, and continues them in persons exposed to infection for several weeks. Carne Ross has lately appealed for an extended trial of cinnamon.[28] He orders half an ounce of decoction of cinnamon every hour for 24 hours, and then every two hours till the temperature falls to normal, after which it is given four times a day for three days. The throat is swabbed every two or three hours with the decoction. The treatment, however, must be commenced as early as possible, at latest within twenty-four hours from the first rigor. Caiger, in Allbutt's system, reports that in two hundred cases treated in this way, though the general death-rate was unaltered, the incidence of complications such as rheumatism, adenitis, and nephritis was reduced by half. Many of these cases were, however, not treated so early as Ross requires, since they are rarely admitted into a fever hospital until the rash has appeared. As a help towards the early diagnosis of scarlatina, we may refer to some points discussed by Lindsay.[29] He holds it to be a good rule to suspect this disease when vomiting, with a sudden rise of temperature, occurs in a child. A very rapid pulse, 140 or 150, is an additional symptom; the rash commences on the chest and neck and wrists, while the face, though flushed, is not attacked early, if at all. In measles and rotheln the rash is seen first on the face. Any one symptom of scarlatina, rash, desquamation, temperature or throat affections may be absent in true cases of the disease. In the diagnosis from rotheln, he relies chiefly on the existence of a prodromal stage, with vomiting, rigors, and malaise, the absence of the rash from the face in most cases, the ulceration of the throat, and absence of enlargement in the post-cervical glands.

D. F. Williams[30] considers the position of the rash immaterial if it is formed of small, bright red spots set close together, but he does not believe that the disease is ever without pyrexia; still the diagnosis must be made from the whole group of symptoms which are present. In the Glasgow Fever Hospital,[31] doubtful cases are placed in separate wards as follows: (*a*) Those where a positive diagnosis of scarlatina is uncertain; (*b*) those where the symptoms suggest another disease, such as tonsillitis; (*c*) cases of obscure illness, such as might turn out to be enteric. Each patient is carefully watched, and if found to have scarlatina he is at once transferred to the special pavilion; the others are detained four weeks, so as to ensure the notice of any desquamation. It has been objected that a patient who ultimately proves to have had the disease is on this plan mixed with those who do not, but no case of infection has arisen in this way. A very large area of air space is allowed to each, and special care is taken in nursing, especially with respect to any discharges. J. O. Symes[32] writes of the secondary rashes which most often appear about the eighteenth to the twentieth day, and may be erythematous, urticarial, eczematous, papular, or even septicæmic. In the latter there is wasting and a high mortality. The others are probably due to an excretion of the products of the disease which normally occurs about that date, and which according to Mahomet is marked by a rise of pulse tension and temperature, albuminuria, and diarrhœa. The writer does not think that the occurrence of these complications is influenced by length of stay in bed, or by the quality of the diet, but the rashes are a useful indication of the existence of the complications.

Alekssjeeff[33] has collected nine instances of post-scarlatinal paralyses. Those which occur at an early stage tend to involve both upper and lower extremities and sometimes cause aphasia, the later ones are hemiplegic, but the prognosis of all is good. Another rare complication is thrombosis of the veins of Galen. Two instances are given by E. W. Goodall.[34] In his own case general convulsions followed by rigidity and coma came on and ended fatally, but there was no middle ear disease, nor was there marked anæmia or more than a slight ulceration of the throat to account for it.

**Tetanus.**—A successful case of the use of anti-toxin is reported by G. R. Turner,[35] but it showed what has been seen in other successful cases—a long incubation and slow development of symptoms in a young patient. The patient was a boy, aged thirteen, who had been wounded in the foot by a rusty nail fourteen days before the stiffness in the jaws appeared, and the attack was not of the severest type. Chloral, indeed, seemed to give more relief than the anti-toxin. Another case under the care of W. Collier[36] was more severe, and died in spite of similar treatment. The period of incubation could not be made out. A. Eddowes (*Lancet*, January 16th) reports a case where recovery followed the injection of 5 minims of a 2 per cent. carbolic lotion twice a day under the skin. The wound had been made about May 19th, suppurated about June 2nd, and trismus was noticed on June 9th. The subsequent progress of the disease was slow, so that there appears some reason to regard it as of the benign type. Kanthack[37] shows that under certain conditions the tetanus bacillus colonies form a close network of filaments. Some of the bacilli are clubbed, and true mycelial forms are common in young cultures. Numerous flagellæ are found all over the bacillus at first, but drop off when the spore-bearing, drumstick stage is reached.

**Small-pox.**—S. Coupland[38] has published elaborate statistics from the epidemics at Gloucester, Leicester, and Dewsbury, showing that (1) where the child population is largely unvaccinated, the attacks and mortality are highest in the earliest years of life; (2) that the mean attack rate is found between 10 and 30 years, but that here the fatality of the attacks is least; (3) that above 30 the lowest rate of attacks is found with a rather higher fatality; (4) that vaccination reduces the incidence of attacks and the fatal results, though in a less degree as age advances; (5) that, though epidemics vary in virulence, the rates are always higher in the unvaccinated than in the vaccinated; (6) the confluent and discrete types of the disease frequently abort in the vaccinated, and severe types are comparatively rare. The *Lancet* in a leading article[39] agrees with Noel Humphreys that if there is to be any relaxation of primary vaccination we must have revaccination made at least equally compulsory. If a conscience clause is admitted, then protection must be made complete by revaccination after ten years for those vaccinated in infancy. Still more is this necessary if official vaccinators are abolished, and a crop of one-mark vaccinees grows up. F. T. Bond[40] urges, in addition, that efficient primary vaccination should be compulsory at the beginning of school life, while the conscience clause should be limited to the case of infants. McVail,[41] at the Epidemiological Society, criticised in detail the statements of the dissentient members of the Commission, and showed their utter weakness, but the paper, though invaluable from its mastery of the entire subject, is too elaborate to be given in abstract.

**Malaria.**—The question whether this is ever a water-borne disease is discussed by Rupert Norton,[42] who remarks that we are never able to trace a whole group of cases to a single source of infection, as those of typhoid often are, nor do we find groups arising in towns where the infection can be traced to water or milk. He shows that in the instances of the French ship "Argo," and in those related by D. Baker, Kershner, and others where the malaria has been attributed to the water supply, either the reports are incomplete or there is much doubt whether the disease was really malaria. While in some stations of the United States' army, where malaria was prevalent, no diminution occurred when distilled water was drunk, still a vast number of practitioners in the Southern States are convinced that malaria is frequently conveyed by water, in which Ross, Helier, and others in India agree. Celli, on the other hand, shows that the introduction of a new water supply into a town did not diminish the malaria, and both he and Salomone-Marino gave large quantities of water from the Pontine Marshes to patients without causing malaria. On the whole, Norton concludes that the weight of proof is against the common belief that malaria can be so conveyed, and notices that in the cases where the supposed disease was clearly water-borne the plasmodium has not so far been found in the blood.

W. G. MacCallum,[44] in discussing the hæmatozoan affections of birds, notices the apparently good health of the birds though marked tissue changes had taken place, and many red corpuscles had been invaded and broken down. Opie also studied the parasites in the red corpuscles of birds at the Johns Hopkins University and found two types, one of which is a rounded body which lies at one end of the cell and the other an elongated one, which lies curved alongside of the nucleus. In many of their processes of segmentation, and in many details of structure they resemble the malarial organism of man, but he considers that they are, however, essentially distinct, and especially show the absence of all movements. Though no discovery has been made as yet as to the life of the human parasite out of the body, Ross[44] has carried out several experiments to prove that the flagellæ are intended to carry on the life of the organism outside the host, and that the crescents which produce them are not degenerate forms, but an important stage in the life-history of the organism, being formed when the density of the blood in which they live is changed to some moderate extent, and not when microbic substances are added to it.

[25] Lancet, Jan. 2. [26] Amer. Jour. Med. Sc., March. [27] Dublin Jour. Med. Sc., Ap. [28] B. Med. Jour., May 1. [29] B. Med. Jour., Feb. 20. [30] B. Med. Jour., Mar. 13. [31] Lancet, Ap. 3. [32] Bristol Med. Chir. Jour., March. [33] B. Med. Jour., Feb. 13. [34] B. Med. Jour., Mar. 30. [35] Lancet, Feb. 6. [36] B. M. J., Feb. 6. [37] Lancet, Jan. 9. [38] Lancet, Feb. 20. [39] Lancet, Feb. 23. [40] Lancet, March 6. [41] Lancet, March 20. [42] Bulletin, Johns Hopkins, March. [43] Ibidem. [44] B. Med. Jour., Jan. 30.

### DISEASES OF THE HEART AND CIRCULATION.

(*Continued from page* 322.)

**Rheumatic and Atheromatous Valve Lesions.**—Handford[10] gives a useful *résumé* of our knowledge of the different varieties of aortic regurgitation, and emphasises the essential difference between those in which the lesion is stationary and those in which it is progressive. The rheumatic cases are characterised by (1) Onset at a much earlier age than when due to atheromatous change; rheumatism is the main cause of aortic regurgitation arising between the ages of five and thirty-five. (2) The arteries are generally sound. (3) The heart muscle is comparatively sound. (4) Apart from fresh attacks the valvular disease soon ceases to be progressive; hence the prognosis is much more favourable than in the atheromatous cases, and the patient may live for many years, even though leading an active life, without any bad symptoms. On the other hand, in the atheromatous cases, (1) the disease is essentially a degeneration, and arises in middle life, rarely before the age of 35, unless of syphilitic origin; (2) the arteries are always involved to a certain extent; (3) the heart muscle is rarely sound; (4) the lesion is almost always progressive; (5) it affects the male much more than the female. There are four chief sub-divisions of atheromatous cases according to the mode of production: (*a*) Syphilitic cases, arising as a rule between the ages of 35 and 50, and affecting sometimes mainly the aorta, and sometimes mainly the valve; (*b*) those arising from laborious work in strong, muscular men between the ages of 45 and 55; (*c*) arteriosclerotic cases, arising between the ages of 50 and 65 in gouty men who habitually eat and drink too much; (*d*) cases arising in old age.

The chief cause of failure of compensation is degeneration of the ventricle from fibroid change, and our chief difficulty lies in the fact that, when this is advanced, digitalis has little action on the heart, and may actually do harm, probably by its vaso-constrictor effect.

**Mitral Disease.**—A question which has arisen lately is, Does a mitral regurgitant murmur developing in the course of rheumatic fever inevitably denote a rheumatic endocarditis of the mitral valve? Dilatation of the left ventricle, if sufficiently great, will certainly produce such a murmur, *e.g.*, in the case of anæmia, which disappears with the improvement of the cardiac condition; and clinical observers are beginning to think that the same condition of affairs is true also of rheumatic fever. The evidence which has recently been brought forward is somewhat as follows: Fisher[11] states that, from examination of post-mortem records, mitral regurgitation pure and simple is a comparatively rare cause of death. Lees[12] finds that in cases of acute or even subacute rheumatism, without either pericardial rub or endocardial murmur, careful percussion will give evidence of the dilatation of the heart, the cardiac dulness may be found to extend one or one and a-half finger's breadth beyond the right edge of the sternum, and the same distance outside the left nipple. At the same time, the first sound may be altered in quality, and weak or muffled. In rheumatism, then, we must admit a direct muscle change that eventuates in dilatation of the heart. If now a mitral regurgitant murmur appears, is it due to this or is it due to the comparatively trivial changes—a mere row of beads—produced (as an initial result) by a rheumatic endocarditis? Steel[13] is inclined to think that cases of pure mitral regurgitation arising from rheumatism are due to muscle failure, and that rheumatism produces either this or mitral stenosis. This theory of muscle failure of the left ventricle in rheumatism offers a simple explanation of the fact that a dilated left ventricle is found in association with a mitral regurgitant murmur. The old explanation was that the regurgitated blood had to be returned to the left ventricle plus the normal quantity, so that the ventricle was, as it were, over-distended during its diastole. Steel further discusses the systolic pulmonary murmur so frequently heard in these cases—both of mitral stenosis and simple muscle cases. It is often associated with a pulsating area in the second left intercostal space close to the sternum. Post-mortem evidence shows that this pulsating area is not the left auricle, as was formerly supposed, but the infundibulum of the right ventricle; and the murmur is produced either in the infundibulum or in the pulmonary artery on the other side of the valve.

In some heart conditions, especially in some cases of mitral stenosis, an apparent double second sound heard only at the apex may be perceived. Phear[14] points out that the term "reduplication of the second sound" has been extended to this, whereas it should be used only to denote a true doubling of the second sound. In true reduplication the doubled sound is most clearly heard at the base, and in many instances is limited to this region; again, the two sounds are heard in very close proximity, and though they may differ in loudness, they are otherwise similar. This condition is a frequent one, and almost certainly due to a synchronous closure of the pulmonary and aortic valves; and the circumstances which produce such a doubling appear to be such as produce a disturbance of the relation which normally exists between the pulmonary and aortic pressures, *e.g.*, high pulmonary tension due to mitral stenosis or mitral regurgitation and other conditions in which the flow of blood through the pulmonary circuit is hindered. It is by no means so commonly found associated with high systemic pressure, but may occasionally appear in cases of chronic interstitial nephritis.

In simulated reduplication the double sound is heard with the greatest distinctness at the apex, an added element follows the second sound as one shifts the stethoscope towards the apex, and this added element follows the true second sound at an appreciable interval, and is different in quality, usually short and abrupt.

Boyd[15] also comes to the conclusion that this reduplication of the second sound at the apex is apparent, not real, and is of opinion that the added element is produced at the mitral orifice; in confirmation of this opinion he notes the fact that this added element may become replaced by a diastolic murmur.

**Pericarditis.**—Pathologists are often aware that pericarditis with effusion is often overlooked clinically. Further, the prognosis in extensive purulent pericardial effusion is very grave, for medical treatment is of no avail, and the results of paracentesis are often disappointing. But insomuch as early and radical operation should increase the percentage of recoveries, it is of great importance to be able to make a reliable diagnosis. Ewart[16] has given numerous practical points to aid one in examination. The diagnosis between pericardial effusion and dilatation of the heart is the great difficulty; but in pericardial effusion the lower angle of the pericardial dulness projects to the right, whereas the right auricular border is convex outwards, and inclines inwards towards the zyphoid below, while the outline of an effusion spreads out at the base. Again, in cardiac enlargement the apex beats at the extreme left limit of the dulness and at its lowest level, whilst in effusion the apex beats somewhat inside and above the boundaries of the dulness. Further, in pericardial effusion there is a patch of marked dulness at the lower left base of the lung extending from the spine for a variable distance outwards; and when all the signs in front support the diagnosis of effusion, the presence of this posterior patch of dulness furnishes a complete and crucial evidence of fluid.

Ewart[17] further points out that pericardial effusion may occur independently of acute pericarditis, under the influence of rheumatism, cardiac affections, and Bright's disease. If of moderate size it may be capable of rapid reabsorption, and in that case, not attracting evidence either by pain or by pressure symptoms of the fluid, may pass unobserved. Rest and diet, with cathartics and diaphoretics, are the only treatment required. In severe cases, however, the effusion may cause pressure signs, and becomes obvious, and in such cases paracentesis may be required.

Dickinson,[18] in discussing the question of adherent pericardium as a cause of fatal enlargement of the heart, points out that the condition is not necessarily associated with any appreciable alteration in size or embarrassment of the heart, and if enlargement occurs it may be due to causes other than the adherent pericardium, *e.g.*, disease of the valves, or lungs or kidneys. Cases in which fatal enlargement of the heart occurs, not susceptible of such possible explanation, are characterised by some common features; they occur in youth only, and as a result of rheumatic pericarditis. Fisher had concluded that the enlargement must be referred to the specially virulent qualities of rheumatic pericarditis. Dickinson, however, says that probably this is merely due to the fact that pericarditis in children, if the very young be excluded, is almost entirely rheumatic; and that probably the sequence of events is that rheumatism causes, quite independently of any valvular lesion, dilatation of the heart, and that the adherent pericardium, so to speak, fixes the heart in this dilated condition, so that it is unable to return to its natural size. The dilatation is the essential mischief.

**Cardiac Therapeutics.**—Russell[19] considers in a most valuable paper the indications and limitations of cardiac therapeutics, more especially in the case of digitalis. He first points out that there are cardiac cases, in which no treatment is required—cases in which compensation is complete, and which come under notice for other disorders. Again, there are cases of cardiac disease, in which cardiac failure is not primary, but dependent on other causes, *e.g.*, indigestion. The important influence which gastric disturbance exercises on organically diseased hearts is not generally recognised.

Cases of aortic stenosis call for therapeutic interference less commonly than any other valvular lesion; but if the left ventricle fails, digitalis is distinctly applicable. When aortic regurgitation exists the question is quite different. If the pulse is from 60 to 70, digitalis is not suitable, for the increase in length of diastole produced is apt to lead to distention of the ventricle; if, on the other hand, the pulse rate is faster, a slowing down of it to about 70 by digitalis is found to be distinctly beneficial. But there are cases in which the pulse is not thus benefited by digitalis, and in these there is often, but not always, other evidence of acute changes proceeding in the endocardium, for it is found, as a general rule, that during the existence of an active endocarditis, digitalis is rather beneficial than otherwise. With a pulse of between 60 and 70, some cases of aortic regurgitation, with heart failure, are markedly benefited by belladonna, say, ♏ 20 of the tincture three or four times a day, which should be stopped immediately the pupil gives the slightest indication of being affected. The explanation of this beneficial action probably lies in the fact that belladonna accelerates the cardiac action, and also relaxes arterial spasm—a point which can readily be appreciated in the radial artery by the finger—and in this relaxation of spasm the coronary arteries share. Following on the relief, arsenic, iron, or other tonics may be given, with occasional resort to belladonna. Iodide of potassium, which so often gives good results in these cases, probably does so by similarly relaxing arterial spasm.

In mitral disease the conditions bear a considerable resemblance to those of aortic disease. Thus in mitral regurgitation digitalis acts admirably when the pulse rate is rapid, it is contra-indicated when the pulse rate has been slowed to 60, and the pulse has become languid, *i.e.*, has a long drawn-out upstroke without a corresponding drawn - out downstroke. Iron and strychnine are then much more useful. In mitral stenosis the relation of heart-rate to treatment is comparable to that in aortic stenosis—the greater the stenosis the slower must be the heart-rate to enable the circulation to be carried on with any degree of efficiency, so that a prolonged diastole is the condition to be aimed at. Hence, if the heart is rapid good results may be expected from digitalis or strophanthus.

**Digitalis.**—That there is a rise of blood pressure produced by the administration of digitalis has long been a well-established fact, but how the rise is produced has been an open question, some believing it to be due entirely to the increased force of the heart, others that it is due to increased arteriole resistance in addition. Brunton and Tunnicliffe[20] have investigated this point by studying the effect on the general blood pressure produced by stimulation of the vagus in digitalised animals, and comparing it with the effect produced on normal animals. In both cases there was, of course, a marked fall of blood pressure, but in the case of digitalised animals the fall was not so great as in normal animals. This lessened fall can only be due to increased arterial constriction. The authors conclude by saying that this vascular constrictive action of digitalis must be taken seriously into consideration in estimating the therapeutic advantages to be obtained from the drug.

**Strophanthus.**—Wilcox[21] says that strophanthus has several well-marked advantages over digitalis as a cardiac tonic: (1) It acts with greater rapidity, often modifying the pulse-rate within an hour. (2) It does not cause vaso-constriction. (3) It has greater diuretic power. (4) It does not cause any disturbance of digestion. (5) It has no cumulative effect. (6) It is of greater value in children and greater safety in the aged. Strophanthus has certainly no such vaso-constrictive effects as digitalis has, and its advantages and disadvantages as compared with digitalis probably depend on this. Thus, as a diuretic one would expect digitalis to act much better, and Wilcox hardly gives sufficient proof of his statement. Again, many men have found strophanthus to be more apt than digitalis to set up gastric disturbance. If the vaso-constrictive effect of digitalis is found to be harmful it can be got rid of by an arterial dilator, *e g.*, iodide, or any nitrite, or nitro-glycerine.

**Nitro-glycerine.**—Murrell[22] says that in the case of nitro-glycerine we should aim not so much at giving a certain dose as at producing a certain pharmacological action. In considering this two points must be kept in mind—the idiosyncrasy of the patient and the rapidity with which tolerance can be established. His rule is to begin with 1 600th grain in the case of women and 1-200th grain in the case of men, and to run up this dose until the patient is taking 1 grain three or four times a day. In cases of angina he gives the 1 per cent. solution, and orders the dose to be made up to a drachm with tincture of capsicum, spirit of chloroform, and peppermint-water; the patient, for emergency work, carries a small bottle of this in each waistcoat-pocket, as it is found by experience that the drug is absorbed much more rapidly when it is kept warm.

[10] Handford, Lancet, March 13, 1897. [11] Fisher, Lancet, July 18, 1896. [12] Lees, Lancet, July 25, 1897. [13] Steel, Med. Chron., Feb., 1897. [14] Phear, Lancet, Jan. 9, 1897. [15] Boyd, Scottish Med. Jour., Feb., 1897. [16] Ewart, March 31, 1896. [17] Ewart, Nov. 21, 1896. [18] Dickinson, Am. Jour. Med. Scien., Dec., 1896. [19] Russell, Brit. Med. Jour., July 25, 1896. [20] Brunton and Tunnicliffe, Jour. of Phys., vol. [illegible]x. [21] Wilcox, Am. Jour. Med. Scien., May, 1897. [22] Murrell, Lancet, Aug. 29, 1896.

